# Spontaneous Hyperinflation of an Intragastric Balloon Causing Gastric Outlet Obstruction

**DOI:** 10.7759/cureus.15962

**Published:** 2021-06-27

**Authors:** Shivanand Bomman, David Sanders, Michael Larsen

**Affiliations:** 1 Gastroenterology, Virginia Mason Medical Center, Seattle, USA

**Keywords:** weight reducton, gastric complications, upper endoscopy, covid 19, adverse reactions, endo bariatrics

## Abstract

A 39-year-old female with a history of obesity (body mass index, BMI = 33.6) had an intragastric balloon (IGB) placed in February 2020 for weight loss. She presented with gastric outlet obstruction after a delay in the removal of her IGB because of the coronavirus disease (COVID) pandemic. Although uncommon, spontaneous hyperinflation of IGBs has been reported and the US FDA also has issued a warning regarding the risk of spontaneous hyperinflation. The etiology of the hyperinflation is unclear, however, gas-forming micro-organism contamination of the IGB fluid has been reported.

## Introduction

Severe acute respiratory syndrome coronavirus 2 (SARS-CoV-2) has disrupted all aspects of health care ranging from primary care [1], cancer care [2], and emergency room management [3]. In March 2020, the Centers for Disease Control and Prevention (CDC) advised to cancel or rebook elective surgeries [4]. Given that bariatric surgeries and endobariatrics are elective, these cases were delayed. Health practitioners in these areas focused on looking after their existing patients and managing the complications of those procedures. Many patients were nervous about presenting to medical attention for routine care. As the pandemic has evolved there are now robust recommendations for the management of bariatric surgery in the context of SARS-CoV-2 [5].

Endoscopic treatments can revise bariatric surgeries to further aid in weight loss or manage complications [6]. Endobariatrics is an evolving field, but the placement of intragastric balloons (IGBs) for weight loss has been available since 1985 [7]. The IGBs work by reducing the luminal size of the stomach and promoting early satiety. These procedures are attractive to some patients in that they are effective, temporary, and have limited complications [7]. This case report summarizes the course of a patient who developed spontaneous hyperinflation of her IGB during the SARS-CoV-2 pandemic.

## Case presentation

A 39-year-old female with a history of obesity (body mass index, BMI = 33.6) had an IGB (Orbera™, Apollo endosurgery Inc., Austin, TX) placed in February 2020 for weight loss. The balloon was filled with sterile saline to a recommended fill volume of 600 mL [8]. She had an excellent clinical response, achieving her intended weight loss goal of 40 lbs. The balloon was to be removed in August 2020 but she did not follow up because of concerns about getting severe acute respiratory syndrome coronavirus disease 2019 (SARS COVID-19) during her scheduled medical visit.

**Figure 1:**
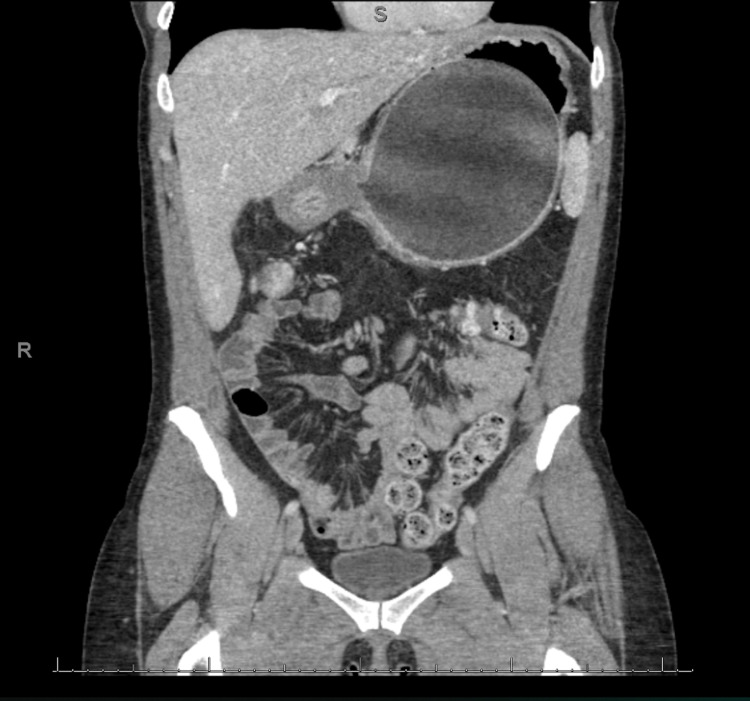
Coronal CT image showing enlarged IGB with stretched gastric wall. IGB, intragastric balloon

**Figure 2:**
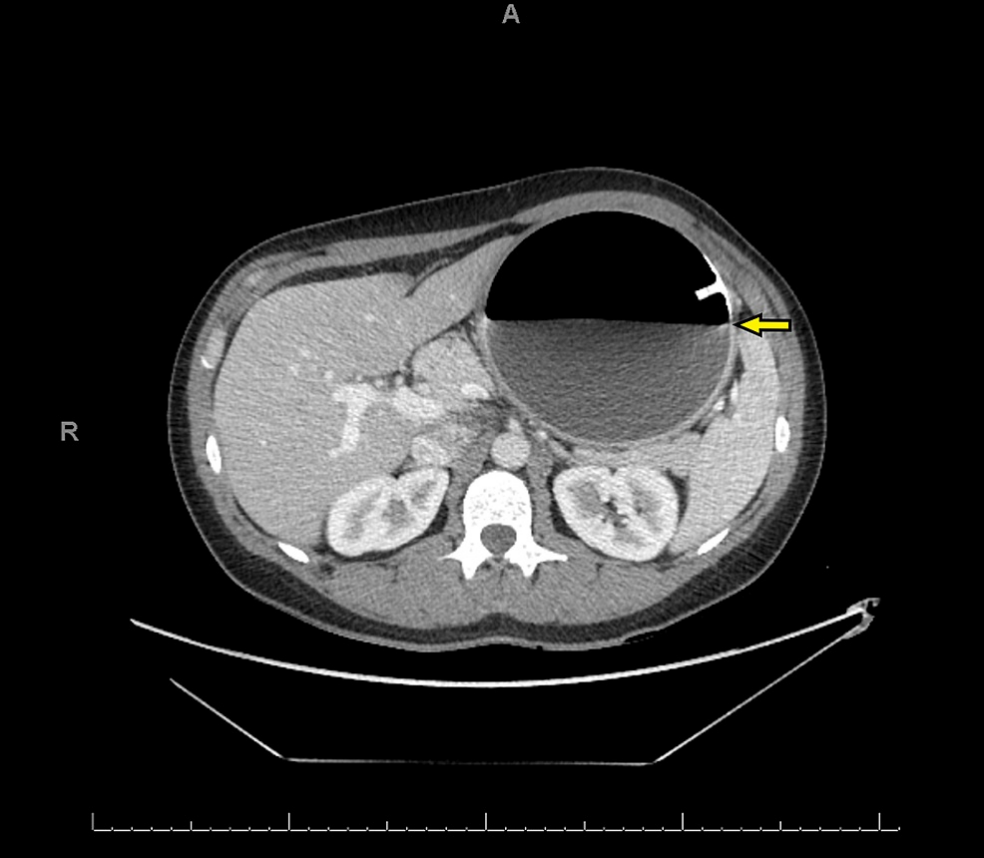
Transverse CT image showing hyperinflated fluid and gas filled IGB. The air fluid level is indicated with an arrow. IGB, intragastric balloon

**Figure 3:**
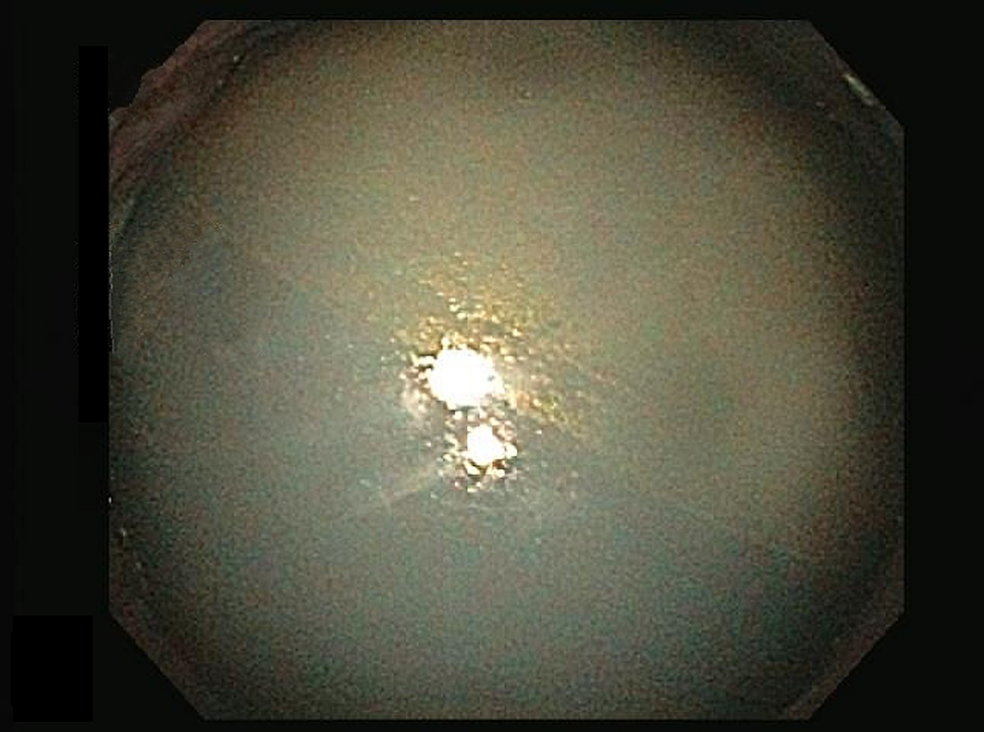
Endoscopic image showing hyperinflated IGB. IGB, intragastric balloon

**Figure 4:**
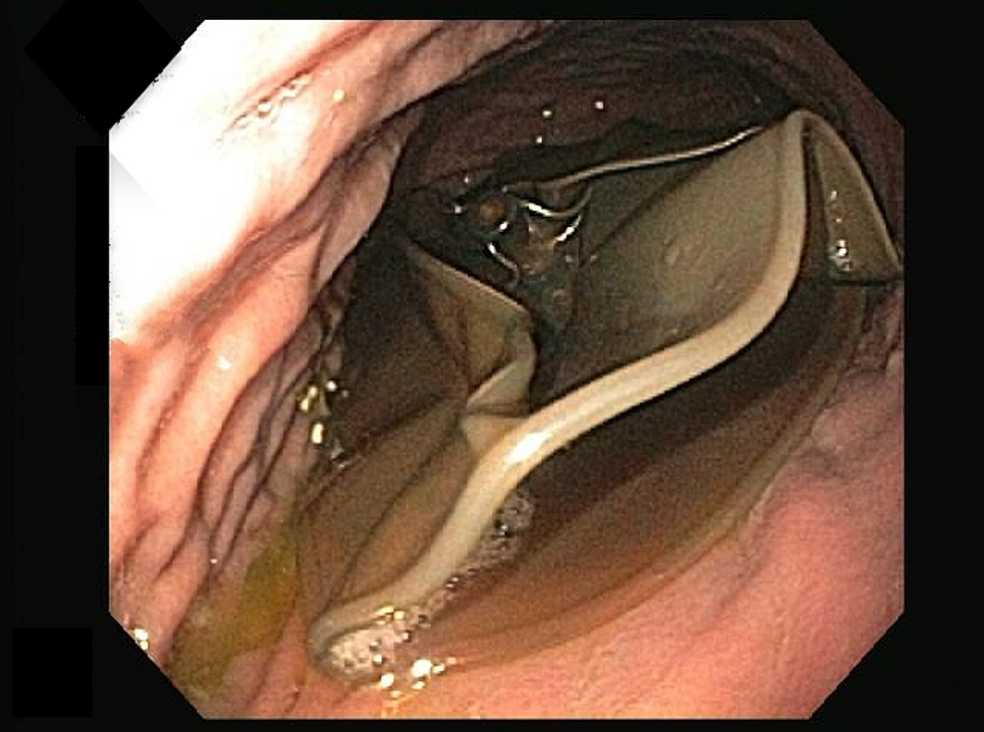
Endoscopic image showing punctured and deflated balloon prior to removal.

She presented to the emergency room two months later with nausea, vomiting, and abdominal pain. A CT scan was performed and demonstrated the IGB within the gastric body and significant gastric distension. The balloon had increased in size and now measured approximately 12.7 cm x 11.6 cm x 13.2 cm (volume of 1011 mL). Half the new volume of the balloon was gas (Figures 1-2). An endoscopy was subsequently performed and a catheter was used to puncture and drain the balloon. Some 600 mL of normal saline was drained and then the deflated balloon was removed without complications (Figures 3-4). The fluid from the IGB was not sent for culture. 

## Discussion

The IGB offers a safe and effective weight loss option for patients with BMI ≥30 and ≤40 [9]. Although uncommon, spontaneous hyperinflation of IGBs has been reported and the US FDA also has issued a warning regarding the risk of spontaneous hyperinflation [10]. The exact mechanism of hyperinflation, in this case, is unclear. Previous reports have found contamination of the IGB fluid with gas-forming micro-organisms like some species of Candida and *Streptococcus viridans* [11-12]. The fluid used in these balloons is sterile and so authors have proposed that the integrity of the balloon can be compromised and become permeable [13]. This patient was not on a proton pump inhibitor. Clinicians can consider sending the fluid aspirated from the balloon for bacterial and fungal cultures. 

Besides gastric outlet obstruction, spontaneous hyperinflation can cause pancreatitis, gastric volvulus, and necrosis [14-16]. This case demonstrates the downstream consequences of SARS-CoV-2 on elective procedures in bariatric care. In this case, the gastric outlet obstruction did not result in any irreversible untoward effects. Suspicion for spontaneous hyperinflation with prompt identification and removal of the balloon is necessary for patients with obstructive symptoms to prevent adverse events like perforation or ischemic gastric injury.

## Conclusions

Obesity is a risk factor for many complications and health conditions. The Center for Disease Control (CDC) has listed being overweight and obese as risk factors for severe illness in SARS-CoV-2 infections. The significant change to how health services were delivered during the SARS-CoV-2 pandemic disrupted access for patients seeking bariatric care. Many patients elected to avoid primary care or follow-up appointments to reduce their risk of getting an infection. As rates of SARS-CoV-2 infections reduce, our hope is that obese patients can safely return to access evidence-based weight loss options.

The intent of an IGB is to induce early satiety and works in combination with dietary changes, exercise, and behavior modification programs. The fact that the balloon can be removed makes it an attractive option over other more permanent weight loss procedures or surgeries. The maximum placement period for the Orbera balloon is six months. The IGBs are safe and effective procedures, however, a rare consequence of this device is spontaneous hyperinflation with gastric outlet obstruction. This case report demonstrates the consequences of spontaneous hyperinflation after a balloon remained in place beyond the recommended placement period because the patient was concerned about getting a nosocomial SARS-CoV-2 infection.
